# Treatment of visuospatial neglect with biparietal tDCS and cognitive training: a single-case study

**DOI:** 10.3389/fnsys.2014.00180

**Published:** 2014-09-29

**Authors:** Anna-Katharine Brem, Evelyn Unterburger, Irving Speight, Lutz Jäncke

**Affiliations:** ^1^Berenson-Allen Center for Noninvasive Brain Stimulation, Division of Cognitive Neurology, Department of Neurology, Beth Israel Deaconess Medical Center, Harvard Medical SchoolBoston, MA, USA; ^2^Department of Neuropsychology, University Hospital ZurichZurich, Switzerland; ^3^Department of Neuropsychology, Zürcher Höhenklinik Wald ZHW, Rehabilitation CenterFaltigberg-Wald, Switzerland; ^4^Division Neuropsychology, Institute of Psychology, University of ZurichZurich, Switzerland; ^5^Center for Integrative Human Physiology, University of ZurichZurich, Switzerland; ^6^International Normal Aging and Plasticity Imaging Center, University of ZurichZurich, Switzerland; ^7^University Research Priority Program “Dynamic of Healthy Aging,” University of ZurichZurich, Switzerland; ^8^Department of Special Education, King Abdulaziz UniversityJeddah, Saudi Arabia

**Keywords:** transcranial direct current stimulation, visuospatial neglect, rehabilitation, cognitive therapy, stroke

## Abstract

Symptoms of visuospatial neglect occur frequently after unilateral brain damage. Neglect hampers rehabilitation progress and is associated with reduced quality of life. However, existing treatment methods show limited efficacy. Transcranial direct current stimulation (tDCS) is a neuromodulatory technique, which can be used to increase or decrease brain excitability. Its combination with conventional neglect therapy may enhance treatment efficacy. A 72-year-old male with a subacute ischemic stroke of the right posterior cerebral artery suffering from visuospatial neglect, hemianopia, and hemiparesis was treated with biparietal tDCS and cognitive neglect therapy in a double-blind, sham-controlled single-case study. Four weeks of daily treatment sessions (5 days per week, 30 min) were started 26 days post-stroke. During week 1 and 4 the patient received conventional neglect therapy, during week 2, conventional neglect therapy was combined once with sham and once with real biparietal tDCS. Week 3 consisted of daily sessions of real biparietal tDCS (1 mA, 20 min) combined with neglect therapy. Outcome measures were assessed before, immediately after, as well as 1 week and 3 months after the end of treatment. They included subtests of the Test for Attentional Performance (TAP): covert attention (main outcome), alertness, visual field; the Neglect-Test (NET): line bisection, cancelation, copying; and activities of daily living (ADL). After real stimulation, covert attention allocation toward left-sided invalid stimuli was significantly improved, and line bisection and copying improved qualitatively as compared to sham stimulation. ADL were only improved at the 3-month follow-up. This single-case study demonstrates for the first time that combined application of tDCS and cognitive training may enhance training-induced improvements in measures of visuospatial neglect and is applicable in a clinical context.

## Introduction

Neglect is a higher-order, supramodal cognitive deficit, which affects space-related behavior not caused by an elementary sensorimotor deficit (Kerkhoff, 2001), and is mainly caused by lesions in frontoparietal cortical and subcortical networks (Doricchi et al., 2008). Symptoms are heterogeneous and are expressed in different sensory-spatial modalities (visual, auditory, tactile, olfactory). Visuospatial neglect occurs in over 40% of right brain-lesioned patients and in 20% of left brain-lesioned patients (Ringman et al., 2004) and more than 60% of the patients remain impaired after the end of rehabilitation (Carod-Artal et al., 2000; Clarke et al., 2002). Importantly, visuospatial neglect also limits the success of other neurorehabilitative interventions, such as physical and occupational therapy.

Treatment options to date show limited efficiency (Bowen et al., 2013) despite being time-intense. They mostly aim to compensate (e.g., optokinetic stimulation or neck muscle vibration) or substitute functions (e.g., prism adaptation), and few aim to restitute functions (e.g., mental imagery). In the clinical setting, different therapeutic approaches are often combined and individually adapted to the needs of each patient.

In the past decade researchers started to investigate the use of non-invasive brain stimulation in the treatment of neglect. The rationale for using brain stimulation for patients with neglect is based on the “hemispheric rivalry model” proposed by Kinsbourne (1987, 1993). According to this model allocation of visuospatial attention toward both hemifields is balanced by mutual transcallosal inhibition, where each hemisphere competes to direct attention to the contralateral hemifield. Brain lesions disturb this balance, and while the unimpaired hemisphere becomes hyperexcitable, the impaired hemisphere experiences a reduction in excitability. Supporting evidence for this model was first presented by Vuilleumier et al. (1996). They described a patient who suffered from visuospatial neglect after a stroke affecting the right angular gyrus, which however improved after a second stroke affecting the left frontal eye fields. Both of these areas are important for shifting attention via connections with subcortical structures, moreover supporting the assumption of a widespread network subserving spatial attention.

Later studies using brain stimulation methods further supported Kinsbourne's model. Non-invasive brain stimulation methods can be applied to either increase brain excitability in the lesioned hemisphere or reduce hyperexcitability in the unlesioned hemisphere. Several groups applied repetitive transcranial magnetic stimulation (rTMS) during a single session (Oliveri et al., 2001; Koch et al., 2008), or over several days (Brighina et al., 2003; Shindo et al., 2006; Song et al., 2009; Lim et al., 2010; Kim et al., 2013), with the latter leading to improvements for up to 2–6 weeks after the end of treatment. Two studies reported symptom improvement after a single session of transcranial direct current stimulation (tDCS) (Ko et al., 2008; Sparing et al., 2009). Generally, studies using multiple sessions of TMS showed stronger effects that lasted over a longer period of time.

Diagnostic guidelines for neglect are limited (e.g., Duncan et al., 2005; Intercollegiate Stroke Working Party, 2008) and recommend a multidisciplinary diagnostic approach. In order to diagnose visuospatial neglect, various diagnostic tools should be combined with clinical observation, as individual deficits vary greatly between patients. Covert attention measures provide a sensitive tool to assess the impact of visuospatial neglect and are used as the main outcome measure for this study. Posner et al. (1984) stressed the importance of the parietal lobe in covert attention processes, specifically the disengagement operation, when the target is located in the contralateral hemifield. Furthermore, alertness functions are known to draw on a right-lateralized fronto-parietal network (Sturm and Willmes, 2001; Jäncke et al., 2003) and improvements in alertness are thought to contribute to neglect recovery. Alertness was therefore used as a control parameter. We furthermore applied more traditional tests such as line bisection, cancelation, and copying and furthermore assessed visual field deficits and ADL.

The rational to use bilateral tDCS in this study is based on Kinsbourne's interhemispheric rivalry model and the hypothesis that concurrent biparietal modulation would have a stronger and longer-lasting impact on interhemispheric balance than unilateral stimulation. Biparietal stimulation (anode right, cathode left) was applied to concomitantly increase brain excitability in the lesioned right posterior parietal cortex (PPC) and reduce hyperexcitability in the unlesioned left PPC. Furthermore, we hypothesized that combining stimulation with conventional neglect therapy could have synergistic effects and therefore enhance treatment efficacy.

To our knowledge, this is the first study to report the combined impact of repeated biparietal tDCS sessions and cognitive neglect therapy on rehabilitation outcome.

## Materials and methods

### Patient

**Figure 1:**
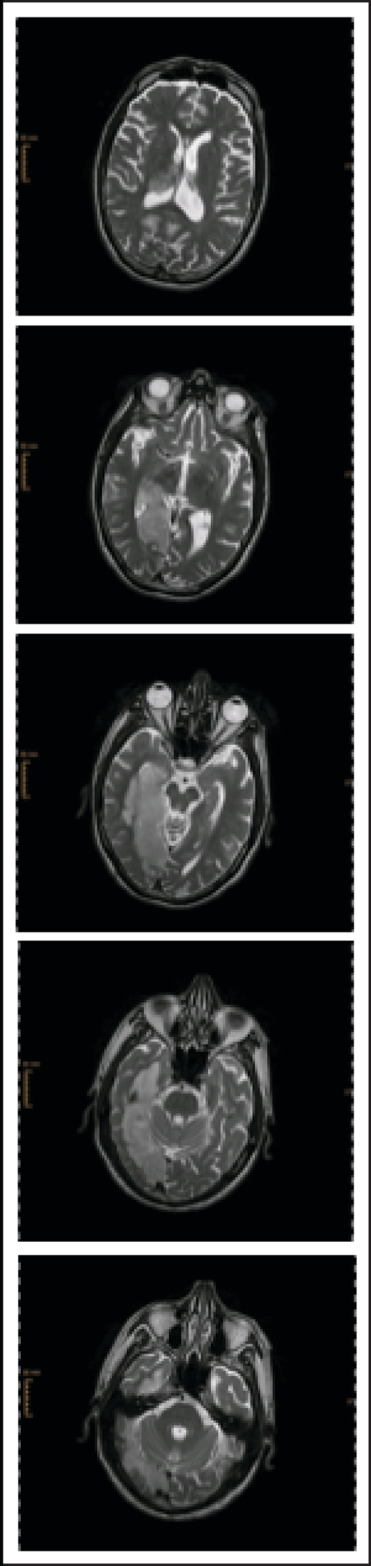
**sMRI T2 after admission to acute hospital**.

We studied a 72-year-old, ambidextrous male who was admitted to the neurorehabilitation unit 23 days after the onset of a moderate ischemic stroke (NIHSS 11/42) of the right posterior cerebral artery of unknown etiology (TOAST 5). At admission he suffered from a left-sided hemiparesis of the arm and face, hemianopia, and severe neglect symptoms. Structural magnetic resonance imaging showed extensive lesions within the right hemisphere affecting mostly subcortical areas of the temporal, parietal and occipital lobe (Figure 1).

Neglect symptoms included sensory as well as motor components (directional hypokinesia). Visuospatial symptoms were more space- than object-centered and N.H. showed a gaze deviation toward the right. Imaginary spatial representation was well preserved as compared to his reduced exploratory-perceptional performance. Alertness performance was initially reduced and fluctuating. Furthermore, impairments in spontaneous speech functions (finding words, dysarthria), and a moderate impairment of verbal and non-verbal memory functions, as well as executive functions (inhibitory control, cognitive flexibility) were observed. On discharge, 12 weeks after stroke onset, he had improved attention allocation toward the left hemi-space, however, transfer to activities of daily living (ADL) was limited, specifically in situations with many external distractors. Executive functions and non-verbal memory improved during rehabilitation but were still impaired at discharge. Verbal memory as well as speech functions normalized. N.H. gave written and oral consent according to the Declaration of Helsinki (1964). Family members were informed about all study procedures and approved of his participation. The protocol was approved by the local ethics committee.

### Study design

**Figure 2:**
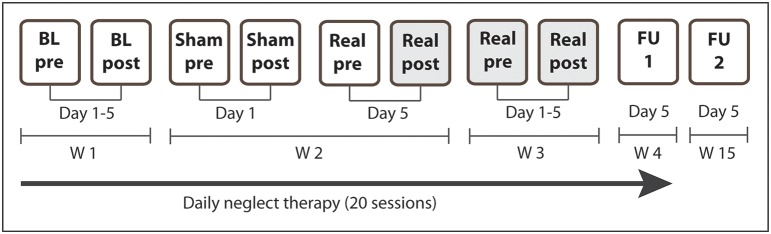
**Study design**. N.H. received daily standard cognitive neglect therapy for 4 weeks (20 sessions). During W1 the patient received standard therapy only (5 sessions). During W2 the patient additionally received sham tDCS on the first day and real biparietal tDCS on the fifth day. During W3 the patient additionally received real biparietal tDCS (5 sessions) and covert attention was assessed after each session. Post-stimulation measures of day 5 (Real post) served as pre-measures for FU1 and FU2. During W4 he received standard therapy only (5 sessions, FU1). Long-term outcome was assessed 3 months after the end of the stimulation (FU2). BL, baseline assessment; FU, follow-up; W, week. Shaded cells depict real stimulation.

This double-blind, sham-controlled single-case study consisted of 4 weeks of daily treatment sessions (5 days per week, 30 min) starting 26 days post-stroke (Figure 2). During week 1 and 4 the patient received conventional cognitive neglect therapy, during week 2, conventional therapy was combined once with sham tDCS and once with real biparietal tDCS. Week 3 consisted of daily sessions of real biparietal tDCS (1 mA, 20 min) combined with neglect therapy and covert attention was measured immediately after every session. Outcome measures were assessed before and after baseline (week 1), single stimulation sessions (week 2), repeated stimulation sessions (week 3), as well as 1 week and 3 months after the end of treatment. After the end of combined treatment, the patient received 7 more weeks of standard neurorehabilitative care before being discharged from the hospital. Assessors and the patient were blinded with regards to stimulation protocols. During the 4-week treatment N.H. furthermore attended occupational (daily), physical (daily), and music therapy (twice per week).

### Assessments

Covert attention, alertness (intrinsic and phasic), and visual field were assessed with subtests of the Test for Attentional Performance (TAP 2.2, Zimmermann and Fimm, 2002). The subtests to assess intrinsic and phasic alertness consist of simple reaction paradigms that require keystrokes as selective reactions to non-verbal stimuli (cross displayed in the middle of the screen). Intrinsic alertness measures the general alertness of the subject, while phasic alertness measures the ability to increase and maintain attention in expectancy of a stimulus. The subtest “Covert Attention” measures the ability to direct visual attention toward a stimulus without change of gaze direction (Posner, 1980) and is a sensitive measure of visual neglect. A central cue (arrow) indicates the side on which a target stimulus (cross) may appear (presentation time of 2 s). The arrow either points in the direction of where the stimulus appears (valid trials) or in the opposite direction (invalid trials). As in invalid trials attentional focus is initially shifted in the opposite direction, re-orientation is needed. Visuospatial neglect is indicated by prolonged reaction times (RTs) in invalid trials toward the contralesional hemi-field. RT to invalid left-sided stimuli was therefore the main outcome measure. Visual field deficits were assessed with the subtest “Visual Field.” In this test peripheral flicker stimuli appear on a black screen (with fixation control) and the subject indicates with a keystroke whether it was perceived.

Line bisection, cancelation, and copying figures were assessed with subtests of the Neglect-Test (NET, Fels and Geissner, 1996), which is a German adaptation of the “Behavioral Inattention Test” (BIT, Wilson et al., 1987). In order to control for transfer effects on ADL, a questionnaire to measure visuospatial disorders (Beobachtungsbogen für räumliche Störungen, BRS, Neumann and Kerkhoff, 2007) was filled out by the occupational therapist and a family member. It measures impairments in ADL such as eating, self-care, dressing, and communication. Furthermore, side effects of tDCS were assessed with a questionnaire.

### Treatment

Computerized training batteries (OK-Neglect: Psycware, Sulzbach-Rosenberg, Germany; RehaCom: Schuhfried GmbH, Moedling, Austria) were presented on a large screen (ca. 1.5 × 2 m) with the patient seated on the right side of the screen. Training was started 5 min after the onset of stimulation and continued for 30 min. Therapies were adapted to the patient's individual needs according to best clinical practice. The main focus was on amelioration of smooth pursuit eye movements (SPEM) and training of saccades toward the neglected hemi-field, as well as visual exploration and reading combined with optokinetic stimulation.

Direct current of 1 mA was delivered for 20 min via two saline-soaked sponge electrodes (7 × 5 cm), which were fixed on the head with a rubber bandage (NeuroConn DC-stimulator, Eldith, Electro-Diagnostic and Therapeutic Systems GmbH, Ilmenau, Germany). The anode was placed over P4 and the cathode was placed over P3 (international 10–20 EEG system). The current intensity used in this study has been used in several publications with stroke patients (Fregni et al., 2005; Hummel et al., 2005, 2006; Hummel and Cohen, 2005; Boggio et al., 2007). In order to achieve comparable sensations for sham stimulation, all sessions started with a slow up-ramping of current over 30 s. At the end of the stimulation current was turned off slowly in order not to elicit perceptions (Gandiga et al., 2006).

### Analysis

The TAP provides norms for adults (TAP 2.2, Zimmermann and Fimm, 2002). Significance levels of intraindividual single-subject differences of *T*-values for the used subtests (intrinsic and phasic alertness, covert attention) were calculated with the software CASE123 (Willmes, 1985, 1990; Guillot and Willmes, 1993) using *T*-values (percent ranks transformed into standard values), test–retest reliability, and subtest correlations. CASE123 is used to perform psychometric single-case analyses according to Huber (1973), which are based on the classical test theory model and are used for tests with standard norms from large standardization samples with satisfactory reliability estimates such as the used TAP. We compared performance pre- vs. post each single session (Sham 1, Real 1, Real 1/5), pre vs. post repeated sessions (Real 5/5), pre vs. post the 1-week control period (FU1), as well as pre vs. post the 3-month control period (FU2). Post-stimulation measures (Real 5/5) served as pre-measures for FU1 and FU2. Paper-pencil test results (line bisection, cancelation, figure copying) are described qualitatively.

In order to evaluate whether coupling stimulation with cognitive training was more effective than cognitive training alone, a 2 × 4 × 3 ANOVA was performed with the within-subjects factors of Time (Pre and Post), Condition (Single Sham, Single Active, Repeated Active, Repeated Training Only), and Test (Invalid Left, Invalid Right, Phasic Alertness).

## Results

### Computerized measures

At admission a complete hemianopia of the left visual field was diagnosed, which persisted until long-term follow up 3 months after the end of stimulation.

**Figure 3:**
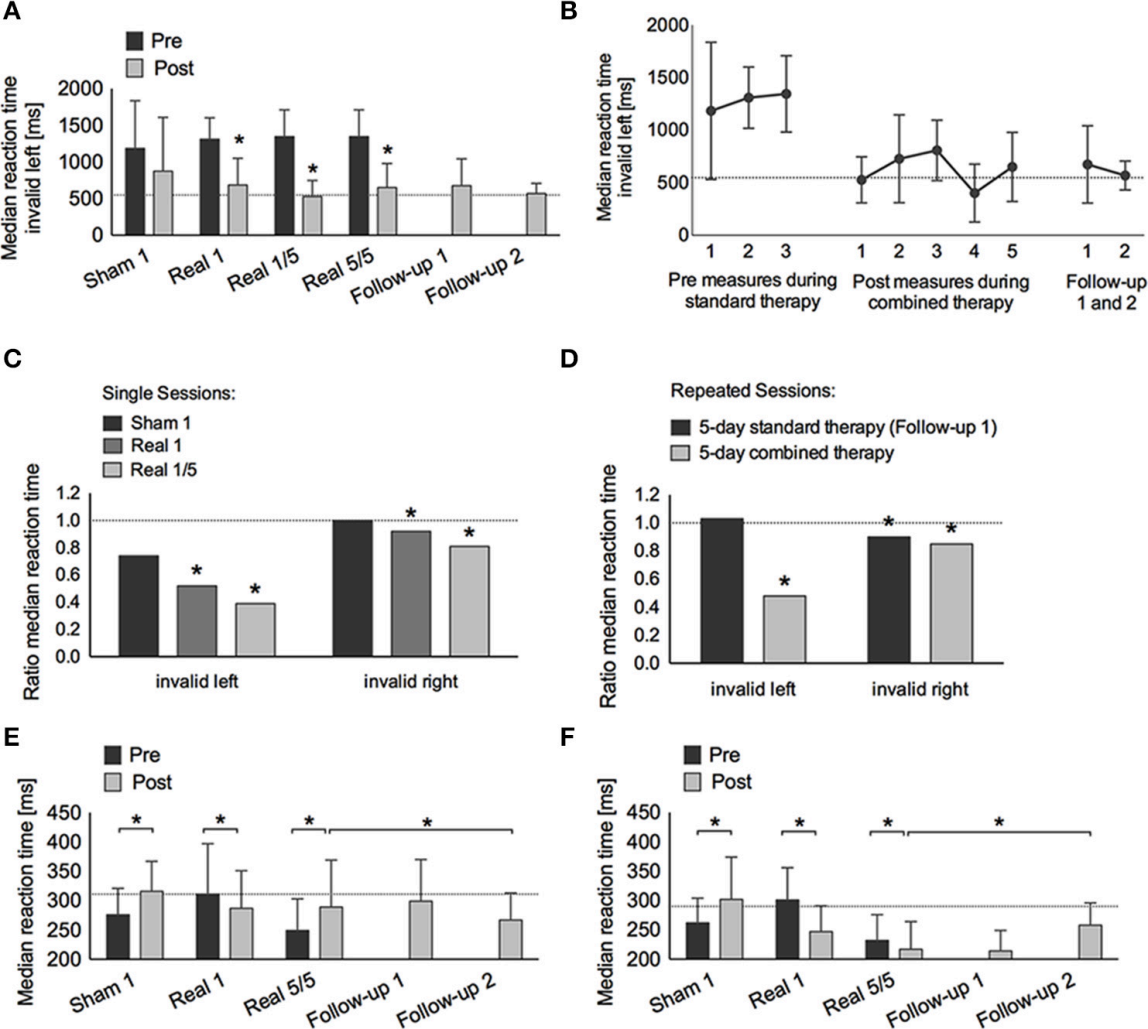
**Covert attention task (Posner paradigm, A–D) and alertness (E,F). (A)** Median reaction time (±*SD*) to invalid left-sided stimuli pre (dark gray bars) and post (light gray bars) treatment: single-sham (Sham 1), single-real (Real 1), single-real on the first day of repetitive stimulation (Real 1/5), repeated-real during 5 days (Real 5/5), standard therapy during 1 week after the end of stimulation (Follow-up 1), and 3 months after the end of stimulation (Follow-up 2). **(B)** Median reaction time (±*SD*) to invalid left-sided stimuli on three different days before real stimulation (pre-measures), after five consecutive sessions with real stimulation (post-measures), and at Follow-up 1 and 2. **(C)** Ratio of median reaction time post-pre to invalid left- and right-sided stimuli after single-sham (Sham 1), single-real (Real 1), single-real on the first day of repetitive stimulation (Real 1/5), and **(D)** after repeated-real during 5 days, and standard therapy during 1 week after the end of stimulation (Follow-up 1). **(E,F)** Median reaction time (±*SD*) in intrinsic **(E)** and phasic **(F)** alertness. ^*^at least *p* < 0.05.

*Intrinsic and phasic alertness* were impaired at admission (intrinsic: *T* = 40; phasic: *T* = 38) with phasic alertness improving significantly after 1 week of standard therapy (*T* = 45, *p* = 0.002). After the single sham session combined with neglect therapy alertness worsened significantly (intrinsic: *T* = 39, *p* < 0.001; phasic: *T* = 38, *p* < 0.001), whereas after the single real session, alertness improved significantly (intrinsic: *T* = 45, *p* = 0.008; phasic: *T* = 49, *p* < 0.001). Both alertness parameters remained within normal levels after the first combined real tDCS treatment (Tables 1, 2, Figures 3E,F).

**Table 1:**
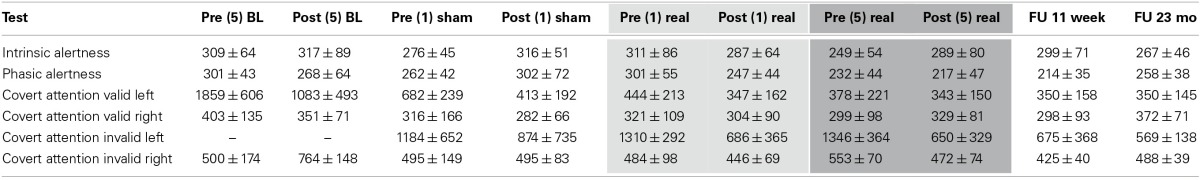
**Raw data alertness and covert attention**.

Abbreviations: BL, baseline standard therapy; (1): 1 therapy session; (5): 5 therapy sessions; FU 1: Follow-up 1 week after the end of stimulation (5 days of standard therapy); FU 2, Follow-up 3 months after the end of stimulation; Mo, months. –: Patient was not able to perceive stimuli. Shading indicates time-periods of combined real tDCS and standard therapy (light gray: single real stimulation; dark gray: repeated real stimulation). Values are presented as median reaction time ± standard deviation.

**Table 2:**
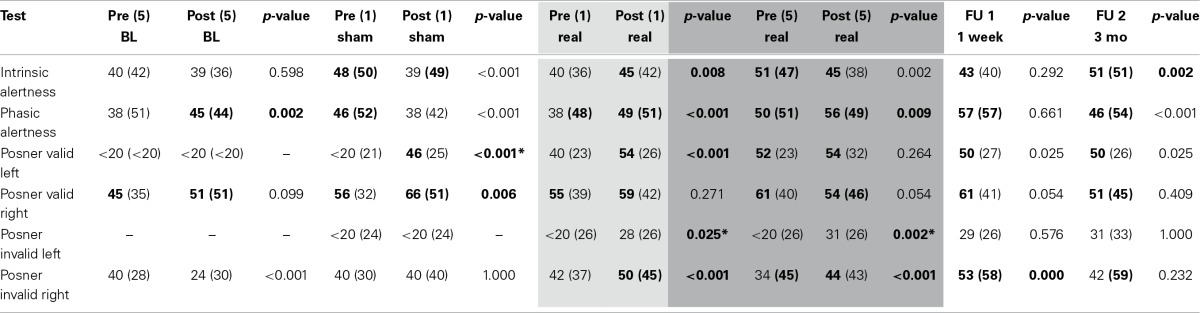
***T*- and *p*-values alertness and covert attention**.

Abbreviations: BL, baseline standard therapy; (1): 1 therapy session; (5): 5 daily therapy sessions; FU 1, Follow-up 1 week after the end of stimulation (5 days of standard therapy); FU 2, Follow-up 3 months after the end of stimulation; Mo, months. –: Patient was not able to perceive stimuli and/or p-values could not be calculated (T < 20). Shading indicates time-periods of combined real tDCS and standard therapy (light gray: single real stimulation; dark gray: repeated real stimulation). T-values of median reaction time and of standard deviations are presented. P-values are two-sided. Bold font indicates performance levels within normal range of the norm population (T-values) or significant improvements (p-values).

^*^For T < 20 p-values were calculated using T = 20, significance of p-values for these time-points are therefore underestimated.

*Covert attention* to *valid right-sided stimuli* was within normal performance levels throughout the study. *Covert attention to valid left-sided stimuli* was strongly impaired at admission, showed significant improvements during standard therapy also reaching a stable and normal level after the first combined real tDCS treatment, and was maintained until long-term follow-up (Tables 1, 2).

*Covert attention to invalid right-sided stimuli* fluctuated at training onset, but stayed below average levels before stimulation onset. After the first single real stimulation it also reached normal levels (*T* = 50). After the weekend on the first day of repeated stimulations RTs had declined again (*T* = 34), but again showed a significant improvement immediately after the first of the repeated stimulation sessions (*T* = 50, *p* < 0.001), remained within normal levels during repeated stimulation sessions (*T* = 44, *p* < 0.001), further improved after the end of stimulation until follow-up 1 (*T* = 53, *p* < 0.001), and returned to post-stimulation levels until the long-term follow-up (*T* = 42, *p* = 0.232) (Tables 1, 2, Figure 3).

*Invalid left-sided stimuli* were not perceived at baseline. By the beginning of week 2 the patient was able to perceive them, however, he showed no improvement after single sham stimulation (*T* < 20, no *p*-value), while significant improvements were observed after a single real stimulation (*T* = 28, *p* = 0.025). Similar as for invalid right-sided stimuli this improvement was not maintained, however, performance improved again even further after the first stimulation session of repeated stimulations (*T* = 43, *p* < 0.001) and remained significantly improved after repeated stimulations (*T* = 31, *p* = 0.002). During the 1-week follow-up with standard therapy performance remained within post-stimulation levels (*T* = 29, *p* = 0.576) and was maintained until long-term follow-up (*T* = 31, *p* = 1.000) (Tables 1, 2, Figure 3).

The main interaction of Time, Condition, and Test was highly significant [*F*_(12, 456)_ = 19.51, *p* < 0.001, η^2^_*p*_ = 0.34]. This interaction was due to a significant interaction for Time and Condition for RTs for left invalid stimuli [*F*_(1, 80)_ = 7.42, *p* < 0.001, η^2^_*p*_ = 0.22], but not for right invalid stimuli [*F*_(3, 64)_ = 0.50, *p* = 0.687, η^2^_*p*_ = 0.23], or phasic alertness [*F*_(3, 312)_ = 2.00, *p* = 0.113, η^2^_*p*_ = 0.02]. The significant interaction for left invalid stimuli was partly explained by a strong trend of the single active condition leading to a larger improvement than the single sham condition [*F*_(1, 40)_ = 4.00, *p* = 0.052, η^2^_*p*_ = 0.09], but was mainly explained by a significantly larger improvement after repeated combined treatment as compared to cognitive training alone [*F*_(1, 40)_ = 21.12, *p* < 0.001, η^2^_*p*_ = 0.35].

### Paper-pencil measures

*Star cancelation* performance fluctuated over the course of rehabilitation and did not vary with stimulation. However, both line bisection and figure copying improved after real stimulation sessions.

**Figure 4:**
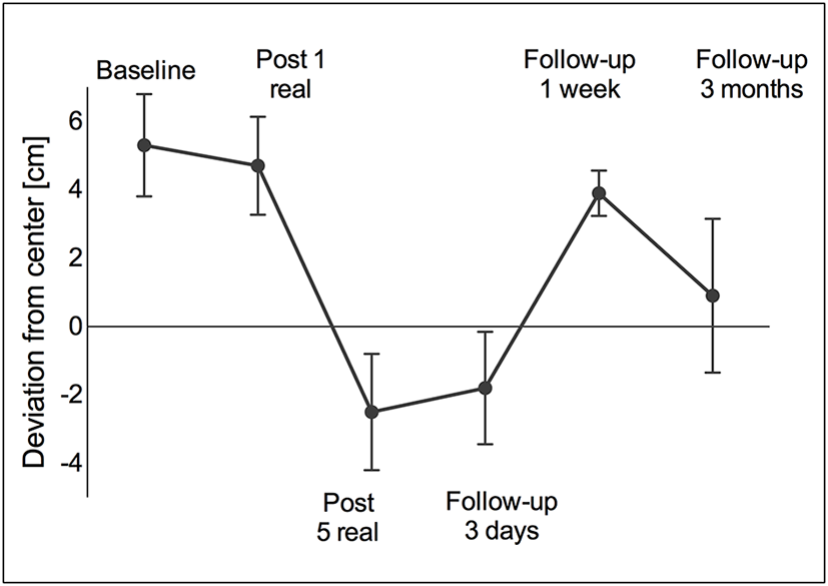
**Line bisection**. A right-sided deviation was observed at baseline and remained right-sided after one real stimulation session. However, after five daily stimulation sessions the deviation turned to the left (over-compensation) and remained left-sided for another 3 days of standard therapy. At the 1-week follow-up as well as at the 3-month follow-up the deviation returned to the right again. Values are presented as mean ± standard deviation (SD).

*Line bisection* was deviated toward the right after the single real stimulation session (5.3 ± 1.5 cm) and before beginning repeated stimulation (4.7 ± 1.4 cm). By the end of the stimulation-week the bisection had deviated toward the left side (−2.5 ± 1.7 cm) and was maintained over 3 days without stimulation (−1.8 ± 1.6 cm). However, by the end of the 1-week follow-up it returned to a rightward deviation (3.9 ± 0.7 cm), but reimproved until the long-term follow-up (0.9 ± 2.3 cm) (Figure 4).

**Figure 5:**
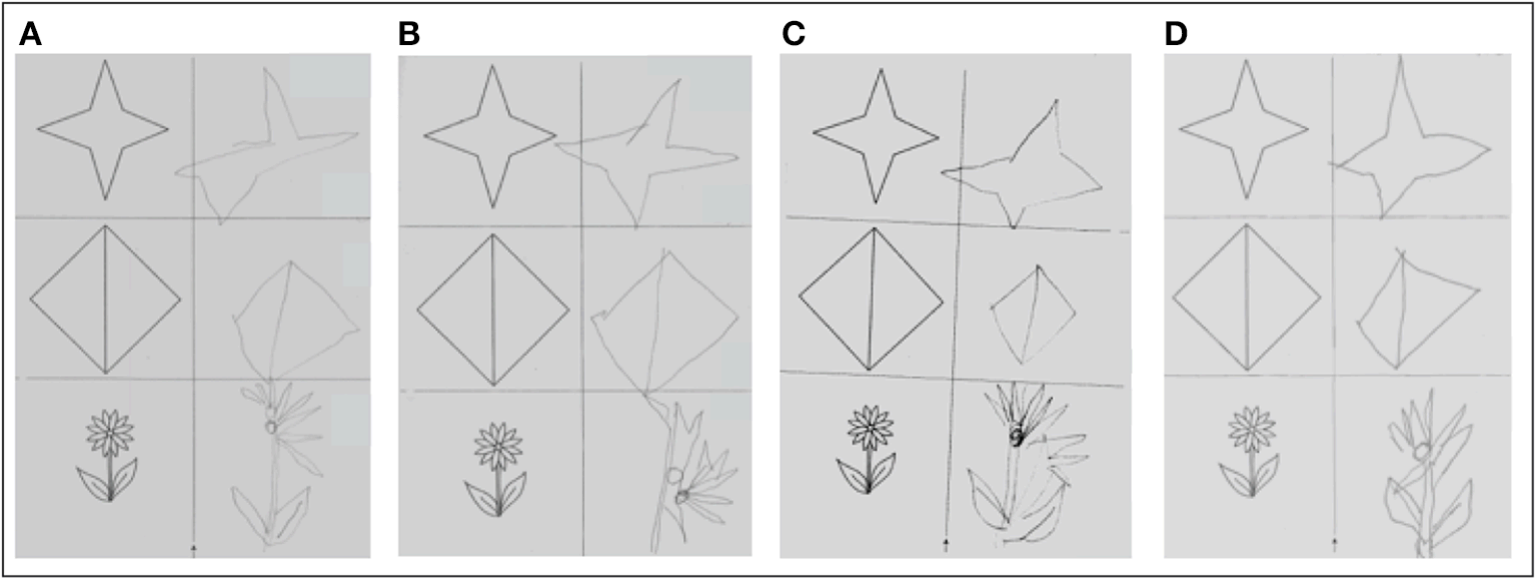
**Figure copying. (A)** Before single real stimulation (5/9 points); **(B)** after single real stimulation (6/9 points); **(C)** before 5 daily real stimulations (6/9 points); **(D)** after 5 daily real stimulations (8/9 points).

*Figure copying* was clearly impaired at baseline (5/9 points). After the single real stimulation it improved by 1 point (6/9 points) and improved another 2 points (8/9 points) after repeated stimulation sessions. However, performance declined again after the end of real stimulation (6/9 points both at the 1-week as well as the 3-month follow-up) (Figure 5).

### Activities of daily living

During his stay at the rehabilitation center, N.H. did not show an improvement in ADL as measured with the BRS (Baseline: 0.9 points; after repeated stimulations: 1.22 points). Only at the 3-month follow-up all parameters showed an improvement (0.16 points).

### Side-effects

The patient reported tingling sensations after the single sham as well as after each of the real stimulation sessions.

## Discussion

This single-case study investigated the long-term impact of repetitive, biparietal tDCS on neglect symptoms in subacute stroke. We found a significantly larger improvement in therapy outcome after combined biparietal tDCS and cognitive training, while cognitive training on its own after single treatment sessions as well as repeated sessions over a time-period of 5 days did not lead to significant changes. Improvements in covert attention as well as alertness were maintained over a follow-up period of 1 week as well as a follow-up period of 3 months after the end of stimulation, whereas performance improvements in paper-pencil tasks were transient, returning to pre-stimulation levels at follow-up.

The main outcome measure (covert attention to left-sided stimuli according to the Posner paradigm) showed the strongest improvement, which was maintained until long-term follow-up, however, without reaching normal levels. Though RTs to invalid stimuli presented in the right hemi-field were also significantly modulated by stimulation, the effects were not as strong as for RTs to left-sided invalid stimuli. When comparing stimulation effects on RTs toward left and right invalid stimuli as well as alertness over the different stimulation conditions (single/repeated, with/without tDCS), only RTs toward left invalid stimuli were significantly modulated by stimulation. This finding supports the assumption of an asymmetrical distribution of brain activity in spatial attention, where the right hemisphere is dominant for orientation of covert attention to either hemi-field (Heilman and Van Den Abell, 1980; Corbetta et al., 1993). On the other hand it corroborates the hypothesis that reducing over-excitability in the undamaged hemisphere can have a beneficial impact.

Alertness measures were used as control measures. After one session of sham tDCS combined with neglect therapy intrinsic and phasic alertness were reduced, which could be due to fatigue. However, after one session of real tDCS, alertness improved significantly. Right frontoparietal areas are specifically important for alertness functions, which might explain why the placement of the anode over the right parietal cortex showed a beneficial impact. Nevertheless, alertness was not the driving factor for the improvement in the RTs toward left-invalid stimuli as we could show that after this initial improvement alertness measures remained within an age-matched norm level and did not show the same pattern of change as RTs toward left-invalid stimuli.

Interestingly, an improvement in most cognitive measures was observed after only one real stimulation session. However, further improvement and stabilization might arise only during repeated and specific combined treatment, which might trigger physiological processes that promote ongoing spontaneous recovery processes. Previous studies found significant long-term changes after only one tDCS session (e.g., Flöel et al., 2012). Effects of tDCS have been associated with NMDA-receptor dependent changes that reflect processes of long-term potentiation (LTP) and depression (LTD) that can become apparent even during or immediately after non-invasive brain stimulation (Liebetanz et al., 2002; Nitsche et al., 2003).

Qualitative performance in the cancelation task was not modulated by stimulation, while performance in figure copying as well as line bisection showed a maximal increase after 5 days of repeated stimulation. In the line bisection task an initially strong ipsilesional deviation, which can be typically observed in neglect patients, turned into a contralesional deviation after repeated tDCS sessions. Such a pattern can be interpreted as over-compensation and occurs usually in patients with hemianopia. Interestingly, over-compensation could still be observed 3 days after the end of stimulation but returned to an ipsilesional deviation at the 1-week follow-up, and finally approached normal levels at the 3-month follow-up. Sparing et al. (2009) also observed a reduction of deviation in a line bisection task but no over-compensation. This effect may be related to the bilateral approach used in the present study. In sum, improvements in paper-pencil tasks were not maintained until the 1-week and 3-month follow-up.

During the rehabilitation period, no measurable transfer of positive effects on ADL was observed. Improvements were only noticeable at the 3-month follow-up. Therefore, it is not possible to determine the association of changes in ADL with stimulation effects. However, in a study by Shindo et al. (2006) most improvement occurred 2–4 weeks after repeated rTMS sessions, which supports the assumption of a kick-off effect of stimulation, which may only later translate into ADL improvements.

It remains to be confirmed in future studies in larger patient samples and including a longer follow-up period that repeated sessions may not only result in a long-term improvement but also have a positive transfer effect on ADL functions. Additional assessments of physiological correlates could furthermore help to elucidate possible delayed stimulation effects.

Several drawbacks of this study need to be mentioned, which are mostly due to the fact that the investigation took place during the subacute stage. First, spontaneous recovery processes need to be taken into account (Cramer, 2008). Furthermore, we cannot rule out the possibility that effects are due to cognitive training alone. Second, the patient participated in other neurorehabilitative programs and practiced by himself. Both of these factors could explain the observed improvements. However, specific effects during the active and sham single sessions contradict these explanations. Furthermore, we would expect continuous improvement over time. Third, some of the paper-pencil tasks could not be assessed at all test-times due to fatigue and time restrictions. Fourth, one could argue that positive effects were caused by a mere improvement of alertness. Though alertness functions might modulate neglect symptoms (Sturm et al., 2006), they do not alone account for this improvement (e.g., after sham stimulation we observe opposite effects on alertness and covert attention). Furthermore, while alertness remained variable within normal levels during repeated stimulation, RTs to invalid stimuli in the covert attention measure improved further. Nyffeler et al. (2009) also suggested that effects were not due to an unspecific increase of alertness, as RTs to right-sided stimuli did not change significantly in their study. We found neither a significant modulating effect of stimulation for alertness nor for invalid right-sided stimuli. Finally, it is in the nature of single-case studies that it is not possible to generalize results to a greater population. Nevertheless, they are helpful in guiding future studies with larger patient populations and can give us valuable insight into specific diseases, providing unique information that might be useful for individualized treatment approaches. Specifically in the subacute stage after a stroke many factors contribute to a large variability, which is a reason why few studies investigate the impact of brain stimulation in subacute patients, although this might be the most fruitful period to stimulate the brain. We may be able to support and guide ongoing compensatory and restitution processes not only through behavioral therapies, but combine them with non-invasive brain stimulation in order to increase beneficial effects.

In sum, during combined therapy functional improvement was significantly higher than during standard therapy alone and was maintained over a follow-up period of 1 week and 3 months. A transfer effect to ADL was only observed at the long-term follow up. Future studies should investigate larger patient populations and longer treatments periods.

## Author contributions

Anna-Katharine Brem and Lutz Jäncke designed the study. Anna-Katharine Brem, Evelyn Unterburger, and Irving Speight contributed to data collection and analysis. Anna-Katharine Brem prepared the draft report, which was critically reviewed by all authors.

### Conflict of interest statement

The authors declare that the research was conducted in the absence of any commercial or financial relationships that could be construed as a potential conflict of interest.
